# RNA editing in disease: mechanisms and therapeutic potential

**DOI:** 10.1261/rna.080331.124

**Published:** 2025-03

**Authors:** Kasra Honarmand Tamizkar, Michael F. Jantsch

**Affiliations:** Division of Cell and Developmental Biology, Center for Anatomy and Cell Biology, Medical University of Vienna, A-1090 Vienna, Austria

**Keywords:** ADAR, interferonopathies, MDA5, RNA editing, site-directed RNA editing

## Abstract

Adenosine to inosine conversion by adenosine deaminases acting on RNA (ADARs) was first identified in the late 1980s of the previous century. As the conversion of adenosines to inosines can be easily detected by sequencing of cDNAs, where the presence of an inosine reads out as a guanosine, the analysis of this type of RNA editing has become widespread. Consequently, several pipelines for detecting inosines in transcriptomes have become available. Still, how to interpret the consequences and alterations of RNA-editing events in whole transciptome editomes is a matter of debate. In particular, the cause or consequence of altered editomes on disease development is poorly understood. Similarly, absolute frequencies of editing events in single molecules, their longitudinal distribution, and naturally occurring changes during development, in different tissues, or in response to physiological changes need to be explored. Lastly, while the use of site-directed RNA editing as a treatment of certain genetic diseases is rapidly evolving, the applicability of this technology still faces several technical obstacles. In this review, we describe the current state of knowledge on adenosine deamination-type RNA editing, its involvement in disease development, and its potential as a therapeutic. Lastly, we highlight open challenges and questions that need to be addressed.

## ADENOSINE DEAMINASE ACTING ON RNAs

Adenosine deaminase acting on RNA (ADAR) enzymes were described as double-stranded RNA (dsRNA) unwinding activities in *Xenopus* oocytes and eggs in 1987 (Bass and Weintraub 1987; Rebagliati and Melton 1987). Later, the activity was attributed to adenosine-to-inosine (A-to-I) RNA-editing enzymes (Bass and Weintraub 1987; Wagner et al. 1989). A-to-I editing is found in almost all metazoa (Wagner et al. 1989; O'Connell et al. 1995; Grice and Degnan 2015). ADAR enzymes bind to dsRNA and hydrolytically deaminate A-to-I. As inosine shares many characteristics with guanosine, this posttranscriptional nucleotide alteration can increase the functional diversity of RNAs. For instance, A-to-I conversion can alter codon identity in mRNAs, thereby dynamically changing the encoded proteome (Li et al. 2009). Likewise, splice sites, RNA secondary structure, and RNA interactions can be affected (Kawahara et al. 2007).

Different types and numbers of ADARs exist in various organismic groups. All ADARs contain a C-terminal catalytic deaminase domain with one to three centrally located double-stranded RNA-binding domains (dsRBDs). The N termini are variable and can harbor functional domains regulating nuclear export and import, Z-RNA-, or single-stranded RNA-binding domains.

**FIGURE 1.**
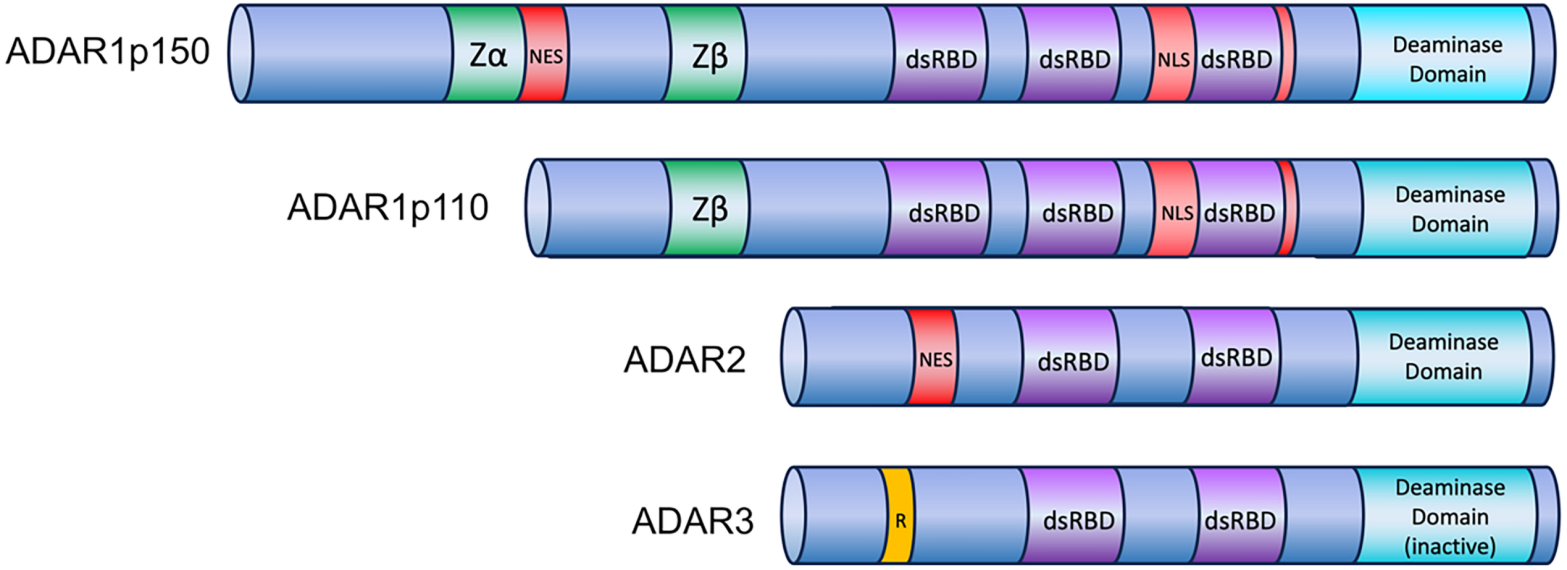
Mammalian ADARs. All ADARs are characterized by a C-terminal catalytic domain. In ADAR3, the deaminase domain is inactive. dsRBDs are required for recognizing double-stranded substrate RNA. ADAR1 has a nuclear localization signal (NLS) flanking its third dsRBD, and ADAR1p150 has an N-terminal nuclear export signal. Consequently, ADAR1 is manly cytoplasmic, while all other ADARs are primarily nuclear. A Zα domain can bind RNAs in Z-conformation in ADAR1p150, while the Zβ domain found in ADAR1p150 and p110 is degenerate.

In mammals, ADAR1, ADAR2, and ADAR3 are found (Fig. 1). ADAR1 has three dsRBDs and N-terminal Z-RNA-binding domains (ZBD). Moreover, it is expressed in two isoforms: Nuclear ADAR1p110 and cytoplasmic ADAR1p150. ADAR1p110 is constitutively expressed, but ADAR1p150 is interferon (IFN)-inducible with a nuclear export signal at the N terminus. ADAR2 is primarily expressed in the nervous system, the vasculature, and the gastrointestinal tract and seems responsible for the majority of editing events that lead to protein recoding (Heraud-Farlow et al. 2017; Jain et al. 2018). ADAR3 is catalytically inactive but may compete for RNA binding with other ADARs (Wang et al. 2019; Raghava Kurup et al. 2022).

## ADAR SUBSTRATES

ADARs can specifically target single adenosines within double-stranded regions of an RNA, while longer dsRNAs can become hyper-edited at multiple sites, as observed in highly complementary inverted Alu-elements and SINEs (Hundley and Bass 2010; Bazak et al. 2014).

A single dsRBD needs at least 15 base pairs of a dsRNA for binding without requiring specific sequence motifs (Polson and Bass 1994; Ryter and Schultz 1998; Lehmann and Bass 2000; Ramos et al. 2000). Still, structural studies have shown that the combination of dsRBDs can help position each other on a substrate and bind ADARs in a distinct orientation (Stefl and Allain 2005; Stefl et al. 2010). Moreover, two dsRBDs can be positioned on opposite sides of a double-stranded A-form helix, thereby allowing dense packing of dsRBDs on relatively short dsRNAs (Lv et al. 2019). ADARs also recognize the end of helices or A-to-I editing-induced contortions, which leads to distinct editing patterns along the RNA double-stranded structure (Lehmann and Bass 1999). Likewise, bulges and mismatches in dsRNAs seem to affect the positioning of ADARs, and, therefore, the symmetry and strand-specificity of edits (Uzonyi et al. 2021; Zambrano-Mila et al. 2023).

## ADARs AND INNATE IMMUNITY

**FIGURE 2.**
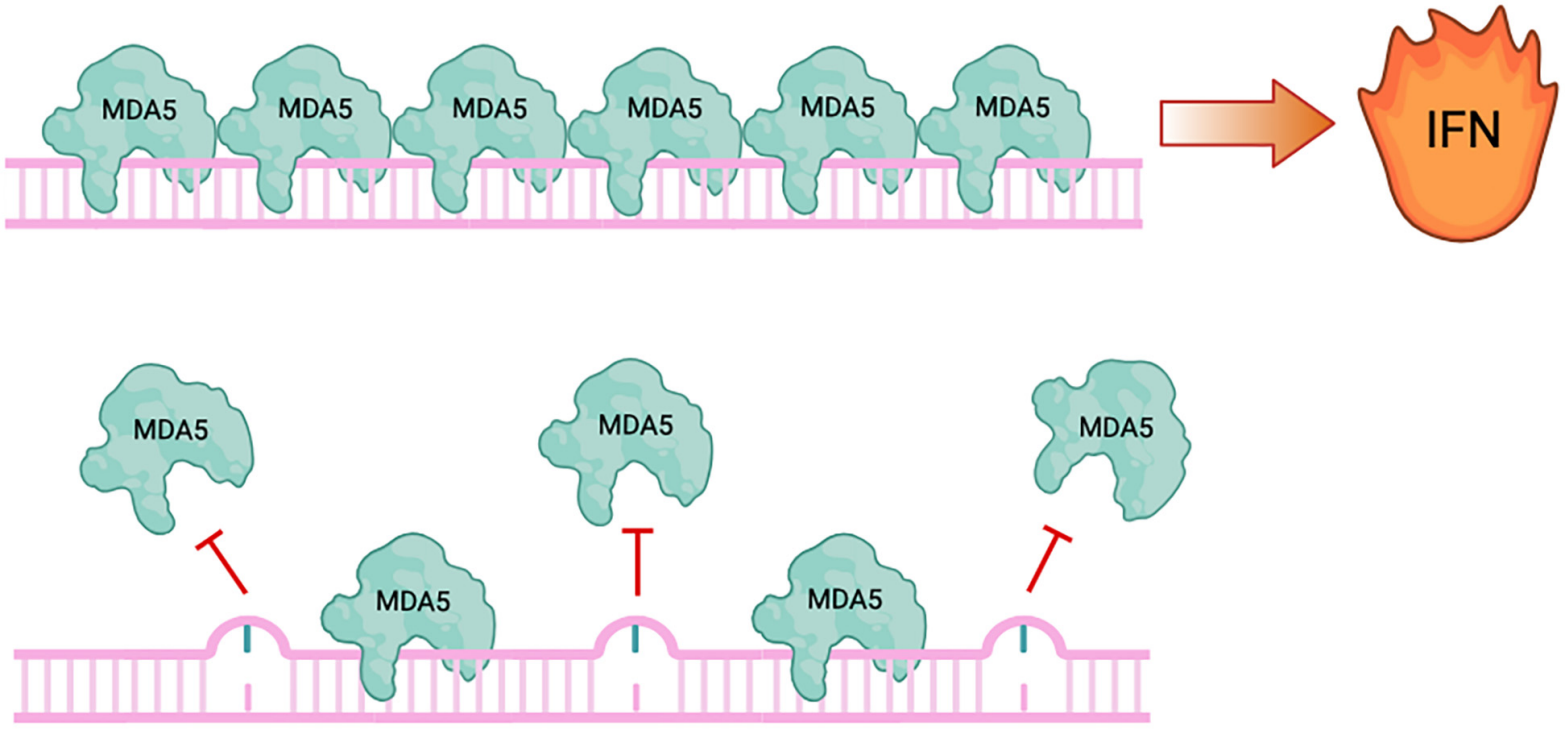
Inosine prevents MDA5 polymerization and activation of IFN production. MDA5 polymerizes along extended, dsRNA structures, which leads to the activation of type I IFNs. This happens mainly along viral dsRNAs to initiate an antiviral defense program. Endogenous RNAs are normally edited by inosines, which prevents MDA5 polymerization.

ADAR1 deficiency leads to embryonic lethality in mice around day 12.5 of development (Hartner et al. 2004). Widespread stress-induced apoptosis of liver hematopoietic cells is accompanied by a strong IFN response (Hartner et al. 2004, 2009; Wang et al. 2004). In humans, mutations in ADAR1 lead to Aicardi-Goutières Syndrome (AGS), an interferonopathy that mimics viral infections during embryogenesis. It was thus speculated that ADAR-mediated editing is required to prevent the activation of cytoplasmic antiviral RNA sensors (Rice et al. 2012). The two main cytosolic dsRNA sensors are retinoic acid-inducible gene I (RIG-I) and melanoma differentiation-associated gene 5 (MDA5) (Yoneyama et al. 2004, 2005). Upon binding to dsRNA, these proteins associate with and activate mitochondrial antiviral signaling (MAVS) protein, which ultimately leads to the expression of IFNs and interferon-stimulated genes (ISGs) to help combat the virus (Lamers et al. 2019). Indeed, the concurrent deletion of ADAR1 and MAVS or MDA5 rescues mice from embryonic lethality, proving the activation of MDA5 in the absence of ADAR1-mediated editing (Fig. 2; Mannion et al. 2014; Liddicoat et al. 2015; Pestal et al. 2015).

Interestingly, deletion of MDA5 rescues different ADAR1 alleles to different extents. While a catalytic inactive ADAR1 can be fully rescued by MDA5 deficiency, full deletions of ADAR1 or mutations in the Z-RNA-binding domain of ADAR1 require additional deletion of PKR and/or ZBP1, another protein carrying a Zα domain (Schwartz et al. 2001; Liddicoat et al. 2015; Chung et al. 2018). A series of manuscripts showed that an impaired ZBD of ADAR1 leads to ZBP1 activation, promoting IFN activation independent of necroptosis-related kinases, such as RIPK1, RIPK3, and MLKL. Furthermore, knocking out ZBP1 in mice fully rescues the ADAR1 mutation, indicating that ADAR1 regulates ZBP1 activation and immune response (Jiao et al. 2020, 2022; de Reuver et al. 2022; Goldeck et al. 2022; Hubbard et al. 2022; Zhang et al. 2022).

It was also demonstrated that ADAR1p150 antagonizes protein kinase R (PKR), another dsRNA sensor that phosphorylates eukaryotic initiation factor 2α (eIF2α) upon viral infection (Manche et al. 1992; Cosentino et al. 1995; Jha et al. 2011). In ADAR1-KO cells, activated, phosphorylated PKR was significantly more abundant than in WT cells, suggesting that ADAR1 inhibits PKR activation. Moreover, adding a transcription inhibitor reduces phosphorylated PKR in cells, showing that ADAR1 edits endogenous RNA to prevent PKR activation (Chung et al. 2018). Finally, a direct protein interaction between the dsRBD3 of ADAR1 and the kinase domain of PKR was shown to inhibit PKR activation (Sinigaglia et al. 2024). Thus, ADAR1 can dampen IFN responses by competing with ZBP1 and PKR for binding to (viral) substrate RNAs and by masking RNAs via RNA editing to prevent inadvertent MDA5 activation (for review, see Goldeck et al. 2022).

## IMMUNE-MODULATING ACTIVITIES OF ADAR

Since ADAR1 editing is required to distinguish self and non-self RNA, misregulation of ADAR1 activity can modulate immune responses (Liddicoat et al. 2015). Interestingly, both hyper- and hypo-editing can have adverse effects. Decreased ADAR1 activity can lead to hypo-editing and consequently cause increased production of IFN-α causing AGS (Crow and Rehwinkel 2009; Rice et al. 2012). A G1007R mutation in *Adar* has also been linked to bilateral striatal necrosis and spastic paraplegia (Rice et al. 2012; Livingston et al. 2014). Disruption of *Adar* in pancreatic beta cells can trigger an islet inflammation accompanied by IFN response, resembling type 1 diabetes onset (Knebel et al. 2024).

In contrast, increased ADAR1 levels and elevated Alu editing are observed in rheumatoid arthritis and systemic lupus erythematosus (Roth et al. 2018; Vlachogiannis et al. 2020). Increased editing of Alu elements in the 3′-UTR region of matrix degradation enzyme cathepsin S (CTSS) has also been shown to stimulate the recruitment of the RNA-binding protein human antigen R (HuR). HuR-binding, in turn, stabilizes CTSS RNA and thus can lead to an increase in CTSS protein. In patients with atherosclerotic cardiovascular disease, ADAR1 expression, CTSS editing, and CTSS mRNA levels are increased (Stellos et al. 2016).

**FIGURE 3.**
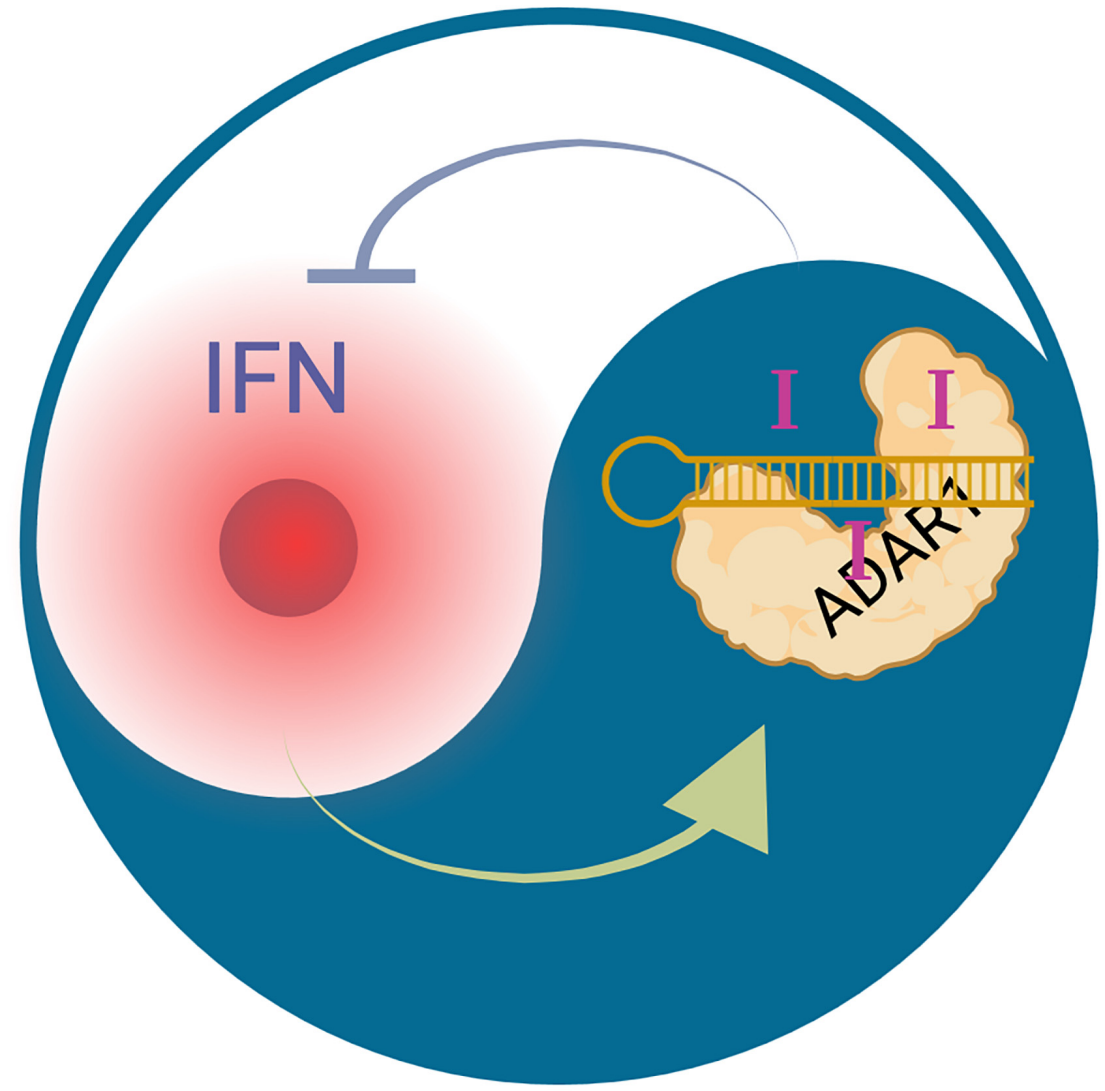
The yin and yang of editing and inflammation. ADAR1 (*Adar*) expression is controlled by type I IFNs. Consequently, inflammatory processes that lead to type I IFN expression will increase the editing signature in cells. Conversely, lack of ADAR1 editing leads to MDA5 activation, which also leads to type I IFN production, which will then turn on *Adar* expression. Therefore, in inflammatory processes, it is difficult to distinguish cause or effect of an inosine signature without understanding the primary causal connections underlying altered inosine signatures in inflamed tissues.

These apparently inconsistent links of ADARs to immune-suppression and immune-activation may be explained by the IFN inducibility of *Adar* (ADAR1) expression. Lack of ADAR1 activity leads to the accumulation of hypo-edited, structured RNAs, which activate the cytosolic dsRNA sensor MDA5, inducing downstream type I IFN expression, as observed in the case of AGS. Conversely, inflammatory processes in rheumatoid arthritis can lead to an IFN stimulus, which then activates *Adar* expression, therefore leading to hyper-editing of some RNAs (Fig. 3).

Consistent with an immune-suppressive function of ADAR1 editing, some cancer cells become dependent on ADAR1 (Gannon et al. 2018) and loss of ADAR1 editing sensitizes tumors to immune checkpoint blockade (Ishizuka et al. 2019). Therefore, ADAR1 has also become a target of interest for tumor therapies with the hope for an immunostimulation caused by ADAR1 inactivation (Bhate et al. 2019; Kung et al. 2021).

## PROTEIN-RECODING ACTIVITIES OF ADARs

While ADAR1 is responsible for the majority of editing events found in repeat regions, nuclear *Adarb1* (ADAR2) seems the enzyme responsible for most protein-recoding events in mammals. As these protein-recoding events alter the functionality of the encoded proteins, they are typically conserved across related or even distant species and seemingly occur only under specific conditions or in certain tissues. Given that a specific dsRNA structure is required to define an adenosine to be targeted, the region frequently involves base-pairing between an exonic substrate-containing region and an adjacent intronic editing complementary sequence (ECS) (Wulff and Nishikura 2010). However, exceptions to this rule do exist (Xing et al. 2023). Of the 100 or so identified editing-induced protein-recoding events only a few have been studied in detail. With no doubt, receptors and their function in the central nervous system are a large group of recoding targets (Tariq and Jantsch 2012; Holmgren and Rosenthal 2015; Rosenthal 2015; Dillman et al. 2016; Gumireddy et al. 2016). However, protein-recoding RNA-editing events also affect secretion, DNA repair, cell division, or cytoskeletal organization (Yeo et al. 2010; Chen et al. 2013; Miyake et al. 2016; Jain et al. 2018). Recoding editing is seemingly most abundant in cephalopods (Liscovitch-Brauer et al. 2017). Alongside, the diversity of ADAR-like enzymes seems more prominent in this organismic group (Birk et al. 2023; Vallecillo-Viejo et al. 2023). Most interestingly, abundant editing in cephalopods may facilitate adaptation to different temperature clines, a phenomenon that had also been observed in *Drosophila* and zebrafish (Rieder et al. 2015; Buchumenski et al. 2017; Birk et al. 2023; Li et al. 2023). Taken together, protein recoding by ADARs can modulate protein function, apparently in response to different stimuli, likely allowing cells and organisms to adapt to internal and external stimuli in a similar way as posttranslational protein modifications can alter protein function.

The above-mentioned examples exemplify that ADARs are involved in different physiological processes that may also lead to diseases, if misregulated. Table 1 lists several pathologies where misregulation of ADARs may be the underlying cause.

**TABLE 1.** Diseases with links to altered ADAR activities

Disease	Description	Reference
Aicardi-Goutières syndrome	Autosomal recessive genetic encephalopathy, varied ADAR1p150 editing leads to MDA5 being triggered by endogenous nucleic acids	Crow and Rehwinkel 2009; Crow et al. 2020
Rheumatoid arthritis	Increased levels of ADAR1p150 expression → increased editing in Alu repeats	Vlachogiannis et al. 2020
Systemic lupus erythematosus	Higher ADAR1 expression but lower ADAR2 levels → increased RNA editing → creation of edited peptides that are MHC class I epitopes → trigger the IFN response	Roth et al. 2018
Diabetes	Endogenous dsRNA/ADAR inhibition in a mouse model → increased IFN response in pancreatic islet cells	Knebel et al. 2024
Bilateral striatal necrosis	A genetic recessive or dominant dystonic movement disorder due to several mutations in ADAR1 such as Pro193Ala, Ile872Thr, and Gly1007Arg	Livingston and Crow 2016
Dyschromatosis symmetrica hereditaria	Autosomal dominant type I interferonopathy → altered pigmentation due to mutations in ADAR1	Miyamura et al. 2003; Li et al. 2010; Kono et al. 2016
Spastic paraplegia	Neurodegenerative, lower limb spasticity, seizures, intellectual disability, Gly1007Arg mutation in ADAR1	Rice et al. 2012

## OPEN CHALLENGES AND FUTURE DIRECTIONS

### Understanding the colinearity of editing patterns

Today, we have considerable knowledge of the possible occurrence of A-to-I modifications in selected cell lines and tissues of model organisms. We also understand some consequences of ADAR1 or ADAR2 deficiency. However, at present, many questions are only partially addressed and will require future work.

**FIGURE 4.**
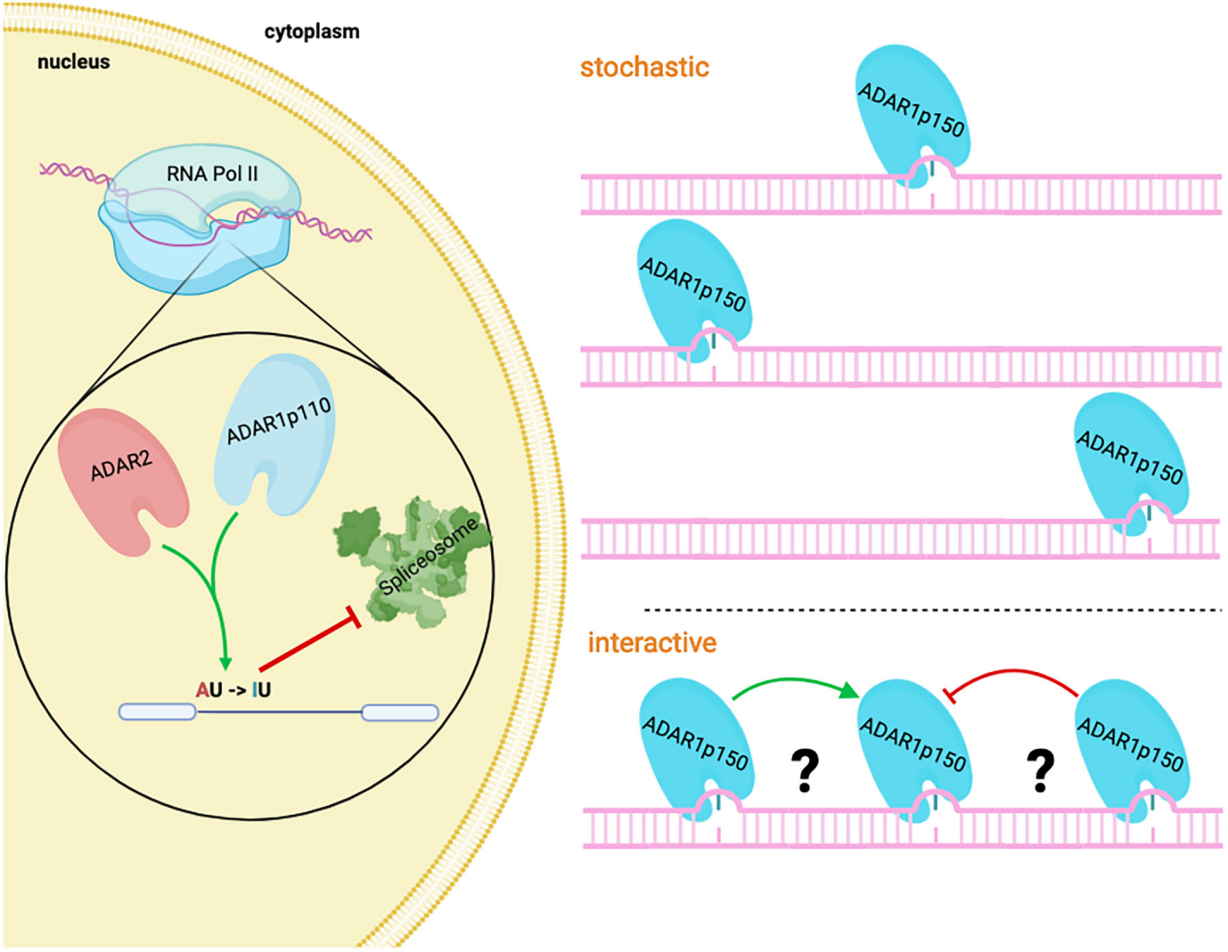
Regulation of inosine deposition. ADAR2 and ADAR1p110 act mainly in the nucleus on pre-mRNA. Recoding events, frequently introduced by ADAR2, are often defined by double-stranded structures formed between introns and exons. Consequently, the splicing machinery and the editing machinery compete for similar regions in RNA and may control each other, as seen in the editing of *Gria2* or *Flna*. Conversely, ADAR can introduce splice sites, therefore introducing novel splice variants. In the cytoplasm, ADAR1p150 is responsible for the hyper-editing of many repeat-derived, structured RNA segments. At present, we do not know whether editing marks are introduced stochastically, on different molecules, or in a coordinated manner along individual RNAs.

For instance, we know little about the linear deposition and distribution of inosines in individual molecules. Whether there is a cascade of modifications happening along stretches of dsRNA or whether one edited position could alter the contiguous structure of the dsRNA and affect editing at flanking positions is not understood (Fig. 4). To achieve this, long-read sequencing technologies must be utilized to study the colinearity of inosines and their distribution per individual RNA molecules. Oxford Nanopore Technologies (ONT) would be well suited to determining the linear arrangement of inosines. However, while several modifications have been successfully detected using ONT (Leger et al. 2021; Begik et al. 2022), the precise calling of inosines on endogenous RNAs still needs some improvement (Nguyen et al. 2022; Chen et al. 2023).

### Camouflaging endogenous RNAs by A-to-I conversion

While we know that the presence of inosines in dsRNAs is critical to suppress the activation of MDA5, the molecular mechanisms preventing MDA5 activation are not understood. This, in turn, comes from the above-mentioned lack of knowledge on the colinear occurrence of inosines along a single dsRNA molecule. MDA5 forms polymers on dsRNA, leading to multimerization of their CARD domains. While it is generally accepted that inosines prevent polymer formation, we do not know whether oligomer formation is completely inhibited or whether MDA5 multimers are destabilized on dsRNAs by the presence of inosines (Fig. 2). Moreover, the stoichiometry of edited versus unedited molecules needs to be understood. Single clone sequencing quite clearly demonstrated that not every molecule is edited to the same extent and some RNA molecules might entirely escape editing. Thus, it is conceivable that editing a few dsRNAs is sufficient to suppress MDA5 activation, possibly in a dominant-negative fashion. However, other models in which a certain threshold of unedited RNAs must be present in a cell in order to activate immune signaling are also conceivable. In any case, understanding the editing patterns and mechanisms that prevent innate immune activation will be imperative to utilize altered ADAR1 editing patterns for therapeutic, immune-activating purposes (Ishizuka et al. 2019).

### Understanding substrate-specific consequences of recoding editing events

**FIGURE 5.**
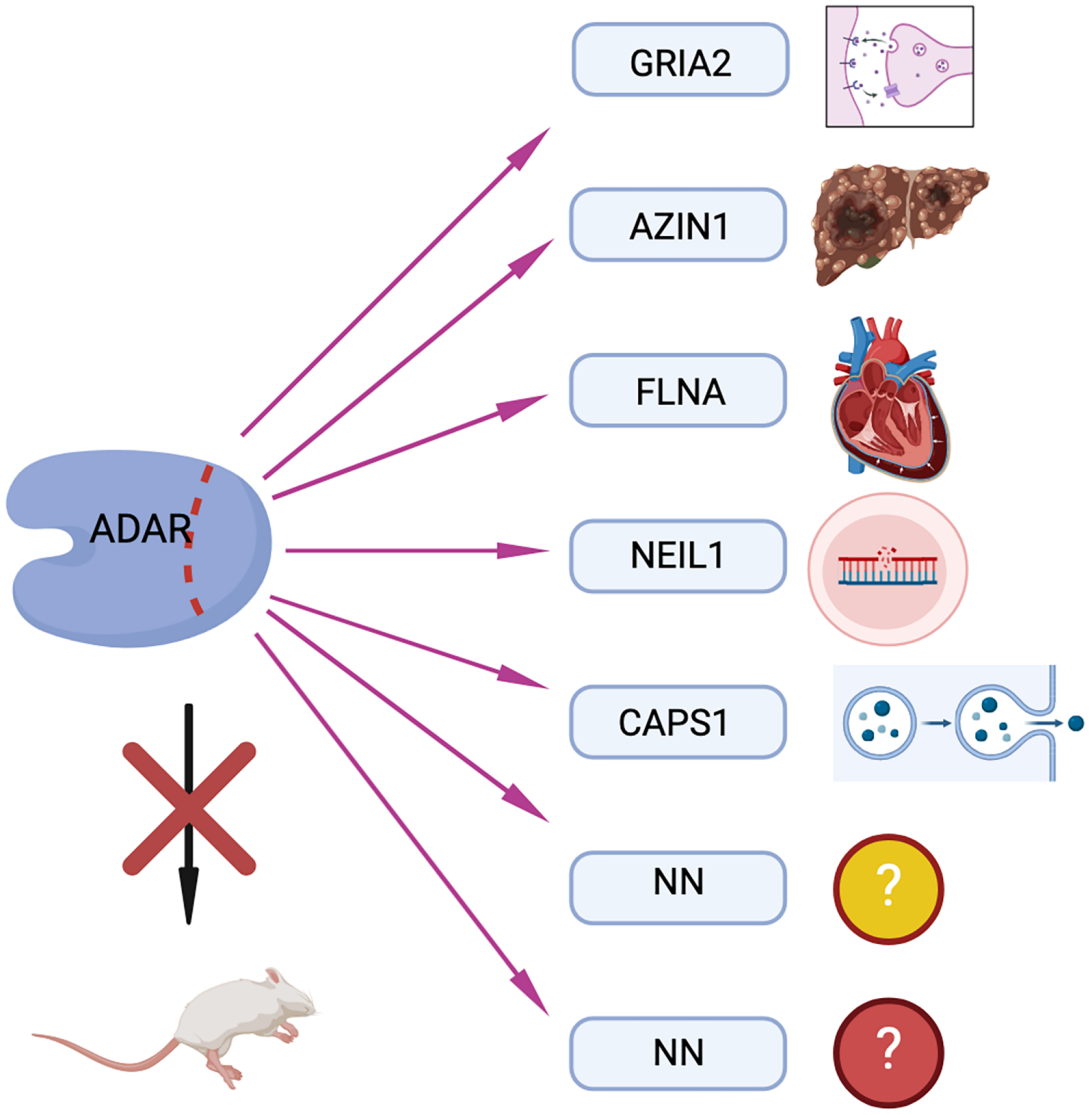
Understanding individual protein-recoding events. ADAR function has so far been mainly studied by investigating the impact of ADAR knockouts in different model systems. Given that each ADAR enzyme has hundreds to millions of editing targets, detailed understanding on the physiological impact on individual (recoding) editing events must be achieved.

As mentioned above, ADAR2 is responsible for most protein-recoding events in mammals. However, the consequences of many protein-recoding events are poorly understood. So far, mainly systemic ADAR2 knockout mice have been studied. Since the postnatal lethality of ADAR2 knockout mice can be well rescued by a preedited version of glutamate receptor subunit *Gria2*, not much attention was paid to the study of other protein-recoding events (Higuchi et al. 2000; Horsch et al. 2011; Miyake et al. 2016; Jain et al. 2018, 2022a,b; Jinnah and Ulbricht 2019). However, it seems evident that highly conserved editing events will have physiological relevance. Therefore, the impact of individual protein-recoding editing events will need to be studied in detail using suitable models under appropriate conditions (Fig. 5; Jinnah and Ulbricht 2019).

### Organismic and physiological variations of editing levels and patterns

Illumina-based sequencing has led to the detection of inosines in selected tissues and cells typically derived from mice, humans, and a few selected model organisms. However, given that RNA editing is a dynamic process, it can be expected that the extent of ADAR-mediated editing will vary between organisms, tissues, and physiological conditions. So far, dynamic, temperature-dependent editing has been shown to occur in cephalopods (Birk et al. 2023). Also, (mainly protein-recoding) editing events will likely respond to physiological changes. In fact, many protein-recoding editing events found in vertebrates are developmentally regulated (Wahlstedt et al. 2009; Veno et al. 2012; Stulic and Jantsch 2013; Czermak et al. 2018). However, we do not understand the underlying mechanisms controlling the extent of editing, nor do we know which other physiological parameters will affect RNA editing.

While it is speculated that the expression of ADARs correlates with the extent of editing in some cases, this is not always true, as illustrated by the example of Filamins (see below). In any case, understanding the conditions that control both recoding and synonymous editing events will improve our understanding of the physiological relevance of editing.

### Filamin editing, a paradigm for site-specific regulation of RNA editing

Filamin A (FLNA) editing leads to a conserved CAG to CIG modification in vertebrates (Stossel et al. 2001; Levanon et al. 2005). In patients suffering from cardiovascular disease, FLNA editing is significantly reduced. Likewise, in a mouse model, impaired editing heightens vascular contraction, diastolic hypertension, and myosin light chain phosphorylation, ultimately leading to cardiac remodeling (Jain et al. 2018).

Filamins are a group of actin-binding proteins comprising Filamin-A, -B, and -C. The pre-mRNAs encoding Filamins A and B are edited at a CAG (Q) codon in exon 42, giving rise to a Q to R exchange in Ig-repeat 22 of the encoded protein. The editing site is defined by a double-stranded structure formed between exon 42 and the adjacent downstream intron in both pre-mRNAs encoding Filamins A and B. Despite both sites being targeted by ADAR2 (*Adarb1*), editing rates differ for both pre-mRNAs. While *Flna* editing is high in the colon and the cardiovascular system, *Flnb* editing is high in bones, cartilage, and brown fat (Stulic and Jantsch 2013; Czermak et al. 2018). This suggests that the activity of ADAR2 is regulated differently on both RNAs, possibly by other factors and processes competing with ADAR2 (see below) (Fig. 4).

### Interactions with RNA maturation and processing

RNAs are transcribed from different endogenous promoters, contain different amounts of introns, and differ in the content and length of their untranslated regions. Nuclear editing can be done by the nuclear editing enzyme ADAR2, which has restricted expression across different tissues, and nuclear ADAR1p110, which is rather ubiquitously expressed (Nishikura 2016).

Thus, for editing events that occur in the nucleus, the dynamics of nuclear processing events and the nuclear dwell time of an RNA will affect the interaction of nuclear ADARs with substrate RNAs. In this respect, RNA-editing events that lead to protein recoding are frequently defined by base-pairing of intronic and exonic sequences (Higuchi et al. 1993; Maas et al. 1996). Therefore, the speed of splicing, which in turn is governed by the assembly of the splicing machinery, its association with the CTD of pol II, and many other factors, will inevitably control editing efficiency. In *Gria2*, an essential editing site in exon 11 is defined by base-pairing with the downstream intron 11. Editing leads to the conversion of a CAG (Q) to a CIG (R) codon, which controls Ca^2+^ influx, preventing cell damage (Verdoorn et al. 1991). Interestingly, the mature (spliced) version of *Gria2* mRNA is edited to 95%. In contrast, editing levels in the pre-mRNA only reach 60%–70%. This and several similar examples suggest that editing can affect the splicing rate, therefore ensuring appropriate editing levels (Szabo et al. 2024). However, the mechanisms underlying these phenomena are only partially deciphered and will require further investigations (Fig. 4).

### Site-directed RNA editing as a therapeutic tool

The fact that protein function can be modulated by recoding-editing has led to various approaches to recruit ADARs to specific sites aiming to alter RNA sequences for therapeutic purposes and to correct pathological mutations at the transcriptome level (Azad et al. 2017). Such site-directed RNA editing (SDRE) can be tunable and reversible and, therefore, may bear fewer risks when compared to genomic engineering using CRISPR/Cas and related methods. For review see (Khosravi and Jantsch 2021).

SDRE methods use antisense guide RNAs to generate double-stranded regions in substrate RNAs, which recruit endogenous or ectopically expressed ADARs to these substrates. While ADARs are used to mediate A-to-I conversions, APOBECs are used in a similar way to introduce C-to-U editing.

For all SDRE approaches, three major challenges exist: (1) durability of the effect, (2) off-target editing, and (3) tissue and cell specificity.

The durability of SDRE not only depends on the stability of the guide RNA but also on the edited template. To this end, guide RNAs can be chemically modified to improve their stability. Numerous chemical modifications have been shown to improve RNA-oligonucleotide stability (Burnett and Rossi 2012; Adachi et al. 2021). Circularization of RNA is another method to increase the half-life of guide RNAs. For instance, gRNAs flanked by twister ribozymes and expressed from plasmids will self-cleave and are subsequently ligated by endogenous RTCB. Such circular gRNAs have been shown to be stable for days (Yi et al. 2022).

Off-target editing can be controlled by optimizing guide RNA sequences and by introducing G:A mismatches opposite off-target adenosines (Booth et al. 2023). Likewise, the introduction of chemical modifications can decrease off-target editing (Merkle et al. 2019).

Still, the biggest obstacle in all RNA-based therapies is the problem of (specific) delivery. While viral vectors have been developed to target several tissues (Wang et al. 2024), GalNac-modified oligos have been shown to specifically target hepatocytes (Benizri et al. 2019; Debacker et al. 2020). Targeting other cell types using specific modifications is a current challenge.

### Therapeutic potential in cancers

As mentioned above, certain tumors become dependent on ADAR1 (Gannon et al. 2018). Likewise, inhibiting editing can help to overcome a PD1 immune checkpoint blockade (Ishizuka et al. 2019). This observation highlights the possibility of a therapeutic approach that could make tumors immunologically reactive by inhibiting ADAR1 activity. However, this would require selective inhibition of ADAR1 in selected (tumor) cells. This bears two challenges: First, inhibitors that selectively block ADAR1 and not ADAR2 would need to be identified, and second, such drugs would need to be selectively delivered to tumor cells, which could be tackled by antibody or receptor-based coupling of ADAR1 inhibitors.

## OUTLOOK

While originally believed to be mainly involved in a few protein-recoding events, ADARs have turned out to be surprisingly versatile modulators of the transcriptome. Several disease-relevant protein-recoding events have been identified in recent years that will require detailed studies to understand their impact on cellular and organismic physiology. Moreover, it was found that ADAR1 can modulate innate immunity through several pathways. Exploiting the multiple functions of ADAR1 may provide useful ways to combat cancer but also to suppress inflammatory reactions. Lastly, site-directed RNA editing has been developed as a new strategy to target genetic diseases in a transient, and therefore relatively safe manner. Without a doubt, a better understanding of ADARs, their regulation, targets, and biological functions will identify additional (disease) relevant functions of A-to-I RNA editing.
